# Self-reported competence among advanced practice nursing students in Denmark, Finland and Norway: a cross-sectional multicentre study

**DOI:** 10.1186/s12912-024-01930-z

**Published:** 2024-05-01

**Authors:** Randi Martinsen, Sigrid Ahlin-Søvde, Ellen Karine Grov, Ewa K. Andersson, Ann Gardulf

**Affiliations:** 1 Department of Health and Nursing Sciences, Faculty of Social and Health Sciences, Section of Advanced Nursing, Inland Norway University of Applied Sciences, Elverum, P.B. 400, Norway; 2 Faculty of Health Sciences, Oslo Metropolitan University, P.B. 4, St. Olavs plass, Oslo, Norway; 3 Department of Health and Caring Sciences, Faculty of Health and Life Sciences, Linnaeus University, Växjö, Sweden; 4 Division of Clinical Immunology and Transfusion Medicine, The Unit for Clinical Research, Department of Laboratory Medicine, Karolinska Institutet, Stockholm, Sweden

**Keywords:** Advanced nursing practice, Advanced practice nursing students, Master’s programmes, NPC Scale-SF, Nurse professional competence scale short-form, Self-reported competence

## Abstract

**Background:**

The health care systems in the Nordic countries and worldwide are under pressure due to increased longevity and a shortage of nurses. Providing nurses with a high level of education, such as advanced practice nursing, is of great importance to ensure effective, safe and high-quality care.

**Aim:**

The aim of this study was to investigate self-reported competence using the Nurse Professional Competence Scale Short-Form for the first time among master’s students of advanced practice nursing in the Nordic countries and to relate the findings to age, work obligations, child-rearing responsibilities, level of education and clinical nursing experience.

**Methods:**

A multicentre, cross-sectional design was used in this study conducted at five universities in Denmark, Finland and Norway. The Nurse Professional Competence Scale Short-Form consisting of six competence areas was used with a maximum score of 7 per item for the analysis of single items and a transformed scale from 14.3 to 100 for analysing the competence areas (higher score equals higher self-reported competence). Descriptive and comparative statistics were used to analyse the data.

**Results:**

The highest mean score was found for the competence area ‘Value-based nursing care’. Students living with home-dwelling children (≤ 18 years) reported significantly higher competence in ‘Nursing care’, while students ≥33 years reported significantly higher competence in ‘Nursing care’ and ‘Value-based nursing care’. No significant differences were found between students working and those not working alongside their studies, between students with and without further nursing-related education, or between students with long and short experience as nurses.

**Conclusions:**

The findings from this study might help to further develop curricula in advanced practice nursing master’s programmes to ensure high-quality nursing and sustainable health care in the future. Future high-quality master’s programmes might benefit from systematic collaboration between Nordic higher education institutions as also Sweden is planning master’s programme. Higher age, having children at home and working while studying should not be considered causes for concern.

## Background

An increase in the population of older people is expected in Nordic countries and worldwide in the coming decades. This demographic trend will represent a challenge for health and medical care in terms of pressure to increase efficiency in health care delivery [1]. Registered nurses (RNs) are viewed as key professionals in health care services and high nursing competence is crucial for patient safety [2, 3]. However, lack of competence has been highlighted as a possible obstacle to achieving or maintaining patient safety and high quality of care, in addition to organizational challenges such as poor continuity of care and inadequate collaboration between care professionals and different service providers. This is particularly prominent in care provision for older people with complex care needs living at home [4].

Continuing professional development is important for the delivery of safe, person-centred and effective care [5]. The World Health Organization has repeatedly highlighted the need for specialist nurses to deal with the global challenge of an ageing population and their health problems. One way to meet these increasing health care demands is to encourage more nurses to take higher qualifications such as a master’s degree in advanced practice nursing (APN) to ensure safe and high-quality nursing care [4, 6] which is also cost-effective [7, 8].

Advanced practice nurses (APNs) are described by the International Council of Nurses (ICN) as RNs who integrate clinical skills associated with nursing and medicine to assess, diagnose, and manage patients in primary health care settings, acute care populations and populations with chronic illnesses. However, the level of practice autonomy and accountability among APNs is determined by and sensitive to the context of the country or setting, as well as the regulatory policies that apply where APNs practice [9]. The ICN states that APNs need to combine knowledge of medicine with a solid foundation in nursing and a nursing master’s degree [9].

Studies reveal that care provided by APNs has positive outcomes for patients [10–14] and reduces unnecessary emergency transfers and hospitalization of older people [15]. Thus, the development of APN master’s programmes is important to meet the future challenges of health care provision [16].

APN students in the context of this study refers to RNs who are attending APN master’s programmes in Denmark, Finland and Norway (120 ECTS). These master’s programmes have similarities in their learning outcomes to the ICN recommendations [9].

Sweden is not included in this study as APN education does not yet exist there, neither as an accredited profession nor as a protected professional title. Over the years, attempts have been made to establish APN study programmes at Swedish universities in close collaboration with local health care providers [17, 18], but Sweden still does not have appropriate national regulations. In 2018, Swedish government officials [19] put forward the need to establish APN education to meet the future challenges of health care, especially for older people with complex needs, and planning for APN education is therefore currently in process.

Qualifying as an advanced practice nurse and transitioning into the APN role means to increase one’s competence, to mature professionally and to gain a broader, holistic view and understanding of care [14]. APN students are expected to improve their decision-making and clinical expertise during their studies [20]. A recent study [21] has pointed out that nurses enrolled in European master’s programmes rate their competence higher than nurses in specialist programmes such as palliative nursing, nurse anaesthesia, intensive care nursing and operating theatre nursing.

Taking professional responsibility for one’s own actions and cooperating in professional teams are nursing skills which APN students rate as being among their highest competencies [22]. However, studies have shown low self-assessed competence in direct clinical practice among APN students [22, 23]. Despite this, self-assessed competence increased considerably during an APN master’s programme, especially for those who assessed themselves as low at baseline [23]. An additional finding by Taylor et al. [23] was that APN students felt that they lacked knowledge in clinical nursing leadership. They also considered that they needed further training in pharmacology and digital competence, such as electronic communication of health promotion and advice [22]. Previous experience of working as an RN and higher education have previously been shown to have no influence on APN students’ self-assessed competence [23].

RNs report a need to increase their nursing knowledge and decision-making in their work [24, 25]. Since it is known that lack of professional development is a contributing factor to RNs leaving their jobs [26], it is urgent to increase professional competence to meet the challenges in health care and society. Professional development is a positive motivating factor for providing high quality of care [5]. During the past ten years, an increasing number of educational institutions in Norway have established APN master’s programmes [27]. In Denmark and Finland, APN master’s programmes have been established within the last five years [28, 29]. The APN master’s programmes run for two full years.

These programmes have not previously been evaluated in terms of students’ self-reported competence and to fill this gap we decided to use the Nurse Professional Competence (NPC) Scale Short Form (SF). This instrument is described in detail in the Method section. The NPC Scale-SF was complemented with background questions. It is important to evaluate the programmes from the students’ perspectives regarding self-reported competence to ensure that the aims of the programmes are fulfilled.

### Aim

The main aim of this study was to investigate self-reported competence among students attending APN master’s programmes in Denmark, Finland and Norway. In addition, we asked questions to ascertain possible differences in self-reported competence in relation to socio-demographic factors such as education after the bachelor’s degree, years of employment as an RN and whether students worked part-time during the APN programme.

## Methods

The study had a cross-sectional multicentre design and was conducted at Danish, Finnish and Norwegian universities. The Strengthening Reporting of Observational Studies in Epidemiology (STROBE) guidelines [30] were used to report the study.

### Sampling

Convenience sampling was used. At the time of this study, four universities in Norway offered APN master’s programmes, but one of these declined to recruit their students to the study. All students enrolled in the other three APN master’s programmes in Norway, the one programme in Denmark and the one in Finland participated (a total of 109 students). The teachers received written and oral information about the study from one of the authors (RM), who also informed them how to present the study orally. The teachers were encouraged to send reminders to the students.

Data were collected during the first year of the APN master’s programme using a survey distributed to the students digitally on their learning platforms from November 2021 to February 2022. The students completed the questionnaires on their computers, cell phones or tablets and submitted their responses in an online survey system developed and operated by the University Information Technology Centre of the University of Oslo [31].

The students received the questionnaire in Danish, English or Norwegian. The reason for using an English version for the students in Finland was that English is the language of instruction in the APN programme there.

### Measurement and scoring

The NPC Scale-SF was published in 2018 and has been shown to be psychometrically sound. It is an instrument to measure self-reported professional nursing competence [32]. The scale has been used for different purposes in several studies including nursing bachelor’s students and nurses attending specialist programmes [32–37]. It consists of 35 items, each beginning with ‘Do you think you have the ability to….’ followed by a question relevant to nursing, e.g. ‘…meet the patient’s basic physical nursing care needs’. The response options range from 1 = To a very low degree to 7 = To a very high degree. When single scores are presented in the results section, they are presented as mean values for this 1–7 grade scale.

The 35 items also form six competence areas (CAs) of importance for nurses’ functions and activities [32]: ‘Nursing care’ (items 1–5), ‘Value-based nursing care’ (items 6–10), ‘Medical and technical care’ (items 11–16), ‘Care pedagogics’ (items 17–21), ‘Documentation and administration of nursing care’ (items 22–29) and ‘Development, leadership and organization of nursing care’ (items 30–35). The scores for each CA are added up, then divided by the highest possible score for the CA (for example, ‘Nursing Care’, consisting of five items, is then divided by 35 [5 × 7 points = 35]) and then multiplied by 100. This gives a scale for each CA with a maximum of 100. The higher the score, the higher the self-reported competence for the CA.

The NPC Scale-SF was complemented by the following background questions: gender, age, living with or without children under 18 years living at home, years of experience as an RN since the bachelor’s degree, any higher education/degree since the bachelor’s degree and paid work or not in health or elderly care while attending the APN programme.

The original NPC Scale was developed in Swedish and has been translated into English in accordance with the WHO recommendations [38]. It has also been translated into Norwegian and the Norwegian version has been validated [39]. The Danish version of the questionnaire was translated for the purpose of this study from English to Danish by a native Danish speaker also fluent in English.

### Statistical analysis

Descriptive statistics were reported with frequencies, percentages and mean, median and SD scores. Additionally, we reported variations with minimum and maximum scores if relevant. The Shapiro-Wilk test showed that the six CAs were normally distributed, and between-group comparisons of continuous variables were analysed with *t*-tests. Cohen’s d was used to measure effect size; 0.2–0.5 small effect, 0.5–0.8 medium effect, 0.8–1.3 large effect, > 1.3 very large effect [40]. Categorical variables were analysed using χ^2^ tests. The level of significance was set at *p* < 0.05 and all tests were two-tailed. When groups were found to be very uneven regarding the number of students, statistics were not performed for gender, other higher degree and part-time work. Data were analysed using SPSS, version 27 [41].

## Results

Thirty-four APN students participated (response rate 31.2%), most of whom were female (88%). Their mean age was 34.1 (range 23–50) years. About two-thirds studied in Norway. Their nursing experience varied from 1 to 20 years, with a mean of 8.2 years. Very few of the participating students (*n* = 3) had a degree from a higher nursing programme other than a bachelor’s degree. Table 1 shows the characteristics of the participants.

**Table 1:** Participant characteristics

Variables	
***Socio-demographic***	
*Age: mean, median, (SD)[minimum-maximum]*	34.1, 33.0, (7.8), [23–50]
*Sex, N (%)*	
Females	30 (88)
Males	4 (12)
*Countries, N (%)*	
Norway	22 (64)
Denmark	6 (18)
Finland	6 (18)
*With children under 18 years living at home, N (%)*	
Yes	14 (41)
No	20 (59)
***Education and experience as an RN, N (%)***	
*Education after bachelor’s degree* ^*a)*^	
Yes	10 (30)
No	23 (70)
*Other higher degree than bachelor’s degree*	
Yes	3 (9)
No	31 (91)
*Experience as an RN in years: mean, median, (SD), [min-max]*	8.2, 6.5, (5.6), [1–20]
***Paid part-time work while studying, N (%)***	
*Working in health or elderly care while studying APN*	
Yes	30 (88)
No	4 (12)
*Part-time dichotomized* ^*b)*^	
≤ 49% of full-time job in health or elderly care	4 (14)
≥ 50% of full-time job in health or elderly care	24 (86)

^a^) Answer from one student missing. Percentage is based on the number of respondents that answered

^b^) Answers from two students missing. Percentage is based on the number of respondents that answered

### Analysis of the six competence areas of the NPC Scale-SF

Table 2 shows the results of the six nursing CAs. The highest mean score was found for the CA ‘Value-based nursing care’. Questions in this CA include e.g. the ability to show respect for different values and faiths, and to show concern and respect for the patient’s autonomy, integrity and dignity. The lowest mean score was found for the CA ‘Care pedagogics’ which asks about the ability to ‘inform and educate patients and next of kin’, and ‘in dialogue motivate the patient to comply with treatments’.

**Table 2:** Students’ self-reported competence based on the NPC Scale-SF. The results are presented as scores (highest competence max 100 score)

Competence Areas	Mean score	SD	Min-max score
Nursing Care	77.6	12.4	51–100
Value-based Nursing Care	82.3	10.8	63–100
Medical and Technical Care	78.5	11.2	52–100
Care Pedagogics	74.5	13.7	57–100
Documentation and Administration of Nursing Care	79.4	11.8	62–100
Development, Leadership and Organization of Nursing Care	74.7	13.2	50–100

The dichotomizing at the median age (33 years) found that students aged 33 years or older (*n* = 16) reported significantly higher competence in the CAs ‘Nursing care’ (*p*-value 0.042) and ‘Value-based nursing care’ (*p*-value 0.022). Furthermore, students with home-dwelling children under 18 years of age reported significantly higher professional competence regarding the CA ‘Nursing care’ (*p*-value 0.017).

No significant differences were found between students with (*n* = 10) or without (*n* = 23) postgraduate education and students with long (*n* = 17) or short (*n* = 17) nursing experience (dichotomized at the median of 6.5 years of nursing experience).

### Analysis of single items on the NPC Scale-SF

The results of comparing the 35 single NPC Scale-SF items between students under 33 years and those aged 33 years and above are displayed in Table 3. Significantly higher scores were reported by the students aged 33 years and above on the items ‘Independently apply the nursing process’ (item 1), ‘Communicate with patients, next of kin and staff respectfully, sensitively and empathetically’ (item 6), ‘Show respect for patient autonomy, integrity and dignity’ (item 7) and ‘Apply emergency medical principles in case of a serious incident’ (item 31) (Table 3).

The students with home-dwelling children under 18 years of age reported significantly higher NPC Scale-SF scores for the items ‘Independently apply the nursing process’ (item 1), ‘Contribute to a holistic view of the patient’ (item 10) and ‘Make use of relevant information in patient records’ (item 22) (Table 3).

Table 3 below shows the scores on the 35 single items in the NPC Scale-SF. The minimum score for the single items was 1 and the maximum score 7 (lowest to highest self-reported competence). The table presents the mean scores, standard deviations (SD) and the statistical results of comparing the mean scores for each item with background data. Regarding age and having home-dwelling children under 18 years of age we found statistical differences but not for the two background factors ‘Years of working as a nurse’ and ‘Any other education after bachelor’s degree’. Items with statistically significant differences in scores are marked in bold. In order to show the higher effect size (ES) calculated for each item, the ES values 0.80 or higher are marked in bold. The names of the 35 items have been shortened in relation to the original wording in the NPC Scale-SF.

**Table 3:** Scores on the single 35 items in the NPC Scale-SF

NPC Single Competence Items	Age ≥ 33 years (*n* = 18) < 33 years (*n* = 16)				Having children under 18 years of age living at home (*n* = 20) or not (*n* = 14)			
Nursing Care, 5 items	Mean score	SD	*p*-value	ES^a)^	Mean score	SD	*p*-value	ES^a)^
1. Independently apply the nursing process	5.67 4.94	1.1 0.9	**< 0.05**	0.74	6.00 4.85	0.9 0.8	**< 0.01**	**1.31**
2. Cater for patient’s physical nursing care	6.11 5.56	0.9 0.8	0.07	0.64	6.14 5.65	0.8 0.9	0.11	0.57
3. Document patient’s physical condition	5.78 5.25	0.9 1.1	0.13	0.53	5.64 5.45	1.0 1.0	0.60	0.19
4. Cater for patient’s psychological nursing care	5.89 5.38	0.8 1.4	0.19	0.46	6.00 5.40	1.2 1.0	0.13	0.55
5. Document patient’s psychological condition	5.11 4.44	1.2 1.0	0.09	0.60	5.21 4.50	1,0 1.3	0.07	0.64
Value-based Nursing Care, 5 items								
6. Respectfully communicate with patients, relatives and staff	6.28 5.44	0.7 1.2	**< 0.05**	**0.94**	6.21 5.65	1.1 0.7	0.10	0.59
7. Show respect for patient autonomy, integrity and dignity	6.28 5.59	0.8 0.8	**< 0.05**	0.77	6.21 5.85	0.9 0.7	0.20	0.45
8. Enhance patients’ and next of kins’ knowledge and experiences	5.72 5.19	0.8 1.2	0.14	0.52	5.71 5.30	1.2 0.8	0.24	0.40
9. Show respect for different values and beliefs	6.06 5.69	0.9 0.8	0.21	0.44	5.86 5.90	0.8 0.9	0.89	0.05
10. Contribute to a holistic view of the patient	5.83 5.25	0.7 1.1	0.08	0.63	6.00 5.25	0.9 1.0	**< 0.05**	**0.84**
Medical and Technical Care, 6 items								
11. Manage drugs and clinical application of knowledge in pharmacology	5.72 5.19	1.0 0.9	0.11	0.57	5.79 5.25	0.9 1.0	0.11	0.57
12. Independently administer prescriptions	5.83 4.88	1.1 1.8	0.07	0.65	5.86 5.05	1.7 1.2	0.13	0.54
13. Pose questions about unclear prescriptions	5.67 5.38	0.8 1.5	0.47	0.25	5.71 5.40	1.3 0.9	0.45	0.30
14. Support patients during examinations and treatments	5.89 5.38	0.8 1.0	0.09	0.60	5.86 5.50	0.9 0.8	0.25	0.41
15. Follow up patient’s condition after examinations and treatments	5.61 5.31	0.9 1.0	0.37	0.31	5.79 5.25	1.0 0.9	0.11	0.57
16. Handle medical/technical products according to legislation and safety routines	5.67 5.25	0.9 1.4	0.30	0.36	5.71 5.30	1.3 0.8	0.31	0.36
Care Pedagogics, 5 items								
17. Provide patients and relatives with support to enhance participation in patient care	5.50 5.31	1.0 1.0	0.59	0.19	5.57 5.30	1.0 1.0	0.44	0.27
18. Inform and educate individual patients and relatives	5.33 5.06	1.1 1.1	0.47	0.25	5.36 5.10	1.0 1.2	0.50	0.24
19. Inform and educate groups of patients and relatives	4.83 4.81	1.5 1.1	0.96	0.02	4.71 4.90	1.2 1.6	0.69	0.14
20. Make sure that information given to the patient is understood	5.44 5.19	0.9 0.9	0.42	0.28	5.43 5.25	0.9 0.9	0.58	0.19
21. Motivate the patient to adhere to treatments	5.33 5.31	1.1 1.1	0.96	0.02	5.36 5.30	1.1 1.0	0.88	0.05
Documentation and Administration of Nursing Care, 8 items								
22. Make use of relevant data in patient records	5.72 5.00	0.9 1.2	0.06	0.69	5.86 5.05	1.1 0.9	**< 0.05**	0.78
23. Use information technology (ICT) as a support in nursing care	5.61 5.06	1.0 1.3	0.17	0.48	5.79 5.05	1.2 1.0	0.07	0.66
24. Document according to current legislation	5.56 5.56	1.2 1.4	0.99	0.00	5.93 5.30	1.3 1.1	0.16	0.51
25. Comply with current legislation and routines	5.67 5.44	1.2 0.8	0.57	0.20	5.86 5.35	1.2 1.1	0.22	0.44
26. Handle sensitive personal data in a safe way	6.11 6.13	0.7 1.0	0.96	0.02	6.29 6.00	0.7 0.7	0.27	0.39
27. Observe work-related risks and prevent them	5.50 5.13	1.0 1.4	0.36	0.32	5.64 5.10	1.2 1.1	0.19	0.47
28. Continuously engage in professional development	5.72 5.88	1.0 1.0	0.67	0.28	5.64 5.90	1.0 1.0	0.47	0.25
29. Lead and develop health staff teams	5.44 5.25	1.1 1.0	0.59	0.18	5.57 5.20	1.0 1.2	0.31	0.36
Development, Leadership and Organisation of Nursing Care, 6 items								
30. Act adequately in the event of unprofessional conduct among employees	5.44 5.06	1.0 1.2	0.31	0.35	5.36 5.20	1.1 1.1	0.68	0.14
31. Apply principles of disaster medicine	5.72 4.88	1.0 1.3	**< 0.05**	0.74	5.79 5.00	1.3 1.0	0.06	0.68
32. Search and review relevant literature for evidence-based nursing	5.17 4.81	1.1 0.8	0.30	0.36	5.36 4.75	0.9 1.0	0.08	0.64
33. Interact with other professionals in care pathways	5.33 5.06	1.1 1.3	0.52	0.22	5.50 5.00	1.3 1.0	0.24	0.42
34. Teach, supervise and assess students	5.44 5.13	1.3 1.3	0.48	0.24	5.50 5.15	1.3 1.2	0.44	0.27
35. Supervise and educate staff	5.50 5.06	1.0 1.3	0.27	0.38	5.36 5.25	1.3 1.0	0.79	0.09

^a)^ ES = Effect size

## Discussion

This multicentre study covered all universities offering an APN master’s programme in the Nordic countries except for one Norwegian university. This was the first time that the NPC Scale-SF were used with APN master’s students. We found a very high internal response rate, which suggests that the items in the NPC Scale-SF were found relevant by the APN students.

We found that the students self-reported high professional competence, especially regarding the CA ‘Value-based nursing care’. This CA also yielded one of the highest NPC scores in a recent study among Polish nurses [42]. In the present study, higher age (33 years or older) resulted in statistically higher self-reported competence for the CAs ‘Nursing care’ and ‘Value-based nursing care’ and the following items within these CAs: ‘Independently apply the nursing process’, ‘Communicate with patients, next-of-kin and staff respectfully, sensitively, and empathetically’ and ‘Show respect for patient autonomy, integrity and dignity’. Students living with children under 18 years of age showed statistically higher self-reported competence for the CA ‘Nursing care’ and the single items ‘Independently apply the nursing process’ and ‘Contribute to a holistic view of the patient’ (the latter was from the CA ‘Value-based nursing care’). As higher education is considered to provide greater professional maturity [14], it would seem reasonable that the life experiences involved in having children and reaching a higher age might enhance communication skills and a holistic approach to patients as described in the items above.

According to our results, additional nursing education or length of work experience prior to entering the APN master’s programme did not influence self-reported competence during the first year of the programme. Taylor et al., using a different questionnaire (ProffNurseSAS II), also found that similar factors had no influence on clinical competence among APN students [22].

The importance of the length of experience as an RN before joining an APN master’s programme has long been discussed among health leaders [37]. It is now important to include in this discussion the new results from the current study, which showed no significant differences between short and long work experience, in addition to results from previous studies [21, 22]. The discussion should include nurse educators active in APN master’s programmes, health authorities and nursing associations, preferably in collaboration between the Nordic countries.

A clear majority of the students performed paid work in health or elderly care while studying, and most worked more than 50% of a full-time job. This is in line with a Swedish national study of 1,086 bachelor’s nursing students, where almost 70% reported having worked in health care during their studies (94.1% of these at least 20 h per week). The students working alongside their nursing education surprisingly reported statistically significant higher competence in all CAs except for ‘Value-based nursing care’ (the latter already had an average score of almost 90 out of 100) [43]. Since working while studying nursing at different levels seems to lead to higher professional competence, concerns about the work-study combination might require less focus in relation to students’ development of professional competence. However, this is a complex topic where other factors, such as students’ total workload, well-being and group dynamics, also need to be taken into consideration by nurse educators and by the students themselves.

Research by Wangensteen and co-workers in a European study found that master’s students assess their clinical competence higher than students in postgraduate nursing programmes [21]. This corresponds with the present study, which found that the master’s students scored significantly higher on the CA ‘Value-based nursing care’ and their ability to ‘Independently apply the nursing process’. This might reflect the likelihood that RNs admitted to master’s programmes are those who are particularly engaged in nursing and knowledge-seeking [44].

In the present study, the third lowest score was for the CA ‘Medical and technical care’. Wangensteen and co-workers [21] also conclude in their study that it is worrying that the master’s students expressed the greatest need for further education regarding medications.

The master’s programmes in Denmark, Finland and Norway have recently been established in line with international standards [9]. However, at the time of this study, there were no ongoing APN programmes in Sweden. A master’s programme in APN with a focus on surgical care is planned to start at one Swedish university, even though there is no national regulation and APN does not exist yet either as an accredited profession or as a protected professional title. There is clearly a need to continue development and collaboration to further establish APN master’s programmes in the Nordic countries, with particular support to establishment in Sweden.

Further research on how master’s students self-report their competence is needed. This study has revealed many interesting results and will now be further developed to investigate master students’ self-reported competence in a longitudinal study. Results from the longitudinal study may provide important knowledge for the curricula of the APN master’s programmes to improve the quality of APN education in the Nordic countries, including Sweden, in the future. The NPC Scale-SF might be one instrument to consider as it has been shown to be sensitive to changes in the curricula of nursing education [35].

### Methodological considerations

Despite great efforts by many people to encourage students to participate in the study, including reminders, the response rate was only 31.2%, which was disappointing. This limitation makes it difficult to draw firm conclusions and comparisons from the results. The first author (RM) was repeatedly in contact with the teachers at the other universities. The teachers received written information about the study, RM informed them how to present the study orally, and we encouraged the teachers to send reminders to participants. We could not contact the students directly via e-mail due to their right to be anonymous. It is known that using online questionnaires generally results in a lower response rate. This has been highlighted by Wu and co-workers [45] in a meta-analysis where they found that online questionnaires on average resulted in a response rate of 44%. Despite all our attempts, the oral information was provided by different teachers, which might have influenced the students’ enthusiasm and interest in the online questionnaire despite reminders. At the time of this study, APN education in Denmark and in Finland was provided at one university in each country, while in Norway four universities had an APN programme. The sample therefore contained a higher proportion of Norwegian students, which must be kept in mind when interpreting the results.

A limitation of self-reported studies is the lack of certainty as to the truthfulness of the responses, as respondents might tend to answer favorably to present a good image of themselves [46]. Moreover, it is not possible to generalize the results of individual self-report studies. However, comparing a study to other studies might strengthen the validity of the study [47]. Consequently, more research on self-reported competence among APN master’s students is needed.

## Conclusion

The NPC Scale-SF was well received by the APN master’s students and they had no comments or questions about the scale, which suggests that they found the questions relevant to their work as nurses and later as APNs. We draw the conclusion that the Nurse Professional Competence Scale-SF could be used for the upcoming longitudinal study.

It is necessary to systematically perform the type of evaluations used in this study in collaboration between Nordic higher education institutions to develop high-quality master’s programmes in APN. The findings from this study might be used to further develop APN curricula to ensure a high level of education and nursing and sustainable health care in the future.

Finally, higher age, having children at home and working while studying should not be considered causes for concern.

## Data Availability

The data will be available from the corresponding author on reasonable request.

## Abbreviations

APN: Advanced practice nursing
APNs: Advanced practice nurses
CA: Competence area
ECTS: The European Credit Transfer and Accumulation System
ICN: International Council of Nurses
NPC Scale-SF: Nurse Professional Competence Scale Short-Form
RN: Registered nurse

## References

[1] Kasai T (2021). Preparing for population ageing in the Western Pacific Region. Lancet Reg Health West Pac.

[2] Church C (2016). Defining competence in nursing and its relevance to quality care. J Nurses Prof Dev.

[3] Aiken LH, Sloane DM, Bruyneel L, Van den Heede K, Griffiths P, Busse R (2014). Nurse staffing and education and hospital mortality in nine European countries: a retrospective observational study. Lancet.

[4] Boman E, Glasberg A-L, Levy-Malmberg R, Fagerström L (2019). Thinking outside the box’: advanced geriatric nursing in primary health care in Scandinavia. BMC Nurs.

[5] King R, Taylor B, Talpur A, Jackson C, Manley K, Ashby N (2021). Factors that optimise the impact of continuing professional development in nursing: a rapid evidence review. Nurse Educ Today.

[6] Delamaire M-L, Lafortune G. Nurses in advanced roles: a description and evaluation of experiences in 12 developed countries. OECD Health Working Papers, No 54. OECD; 2010; 10.1787/5kmbrcfms5g7-en

[7] Craswell A, Dwyer T, Rossi D, Armstrong C, Akbar D (2018). Cost-effectiveness of nurse practitioner–led regional titration service for heart failure patients. J Nurse Pract.

[8] Martin-Misener R, Harbman P, Donald F, Reid K, Kilpatrick K, Carter N (2015). Cost-effectiveness of nurse practitioners in primary and specialised ambulatory care: systematic review. BMJ Open.

[9] International Council of Nurses. Guidelines on advanced practice nursing 2020. https://www.icn.ch/system/files/documents/2020-04/ICN_APN Report_EN_WEB.pdf. Accessed 10 Aug 2023.

[10] Laurant M, van der Biezen M, Wijers N, Watananirun K, Kontopantelis E, van Vught AJ (2018). Nurses as substitutes for doctors in primary care. Cochrane Database Syst Rev.

[11] Chavez KS, Dwyer AA, Ramelet A-S (2018). International practice settings, interventions and outcomes of nurse practitioners in geriatric care: a scoping review. Int J Nurs Stud.

[12] Bergman K, Perhed U, Eriksson I, Lindblad U, Fagerström L (2013). Patients’ satisfaction with the care offered by advanced practice nurses: a new role in Swedish primary care. Int J Nurs Pract.

[13] Morilla-Herrera JC, Garcia-Mayor S, Martín-Santos FJ, Uttumchandani SK, Campos ÁL, Bautista JC (2016). A systematic review of the effectiveness and roles of advanced practice nursing in older people. Int J Nurs Stud.

[14] Wisur-Hokkanen C, Glasberg AL, Mäkelä C, Fagerström L (2015). Experiences of working as an advanced practice nurse in Finland–the substance of advanced nursing practice and promoting and inhibiting factors. Scand J Caring Sci.

[15] Ono M, Miyauchi S, Edzuki Y, Saiki K, Fukuda H, Tonai M (2015). Japanese nurse practitioner practice and outcomes in a nursing home. Int Nurs Rev.

[16] WHO. Transforming and scaling up health professionals’ education and training: World Health Organization guidelines 2013. Geneva: World Health Organization. 2013. https://www.who.int/publications/i/item/transforming-and-scaling-up-health-professionals%E2%80%99-education-and-training. Accessed 31 Aug 2023. 26042324

[17] Jangland E, Becker D, Börjeson S, Doherty C, Gimm O, Griffith P (2013). The development of a Swedish nurse practitioner program– a request from clinicians and a process supported by US experience. J Nurs Educ Pract.

[18] Altersved E, Zetterlund L, Lindblad U, Fagerström L (2011). Advanced practice nurses: a new resource for Swedish primary health-care teams: APNs: a new Swedish health-care resource. Int J Nurs Pract.

[19] Government Offices. *The specialist nurse of the future–new role, new opportunities*. In Swedish: Framtidens specialistsjuksköterska–ny roll, nya möjligheter. Stockholm: SOU 2018:77. https://www.riksdagen.se/sv/dokument-och-lagar/dokument/statens-offentliga-utredningar/framtidens-specialistsjukskoterska-ny-roll-nya_h6b377/. Accessed 15 Aug 2023.

[20] Nieminen AL, Mannevaara B, Fagerström L (2011). Advanced practice nurses’ scope of practice: a qualitative study of advanced clinical competencies. Scand J Caring Sci.

[21] Wangensteen S, Finnbakk E, Adolfsson A, Kristjansdottir G, Roodbol P, Ward H (2018). Postgraduate nurses’ self-assessment of clinical competence and need for further training. A European cross-sectional survey. Nurse Educ Today.

[22] Taylor I, Bing-Jonsson P, Wangensteen S, Finnbakk E, Sandvik L, McCormack B (2020). The self‐assessment of clinical competence and the need for further training: a cross‐sectional survey of advanced practice nursing students. J Clin Nurs.

[23] Taylor I, Bing-Jonsson P, Finnbakk E, Wangensteen S, Sandvik L, Fagerström L (2021). Development of clinical competence–a longitudinal survey of nurse practitioner students. BMC Nurs.

[24] Allvin R, Bisholt B, Blomberg K (2020). Self-assessed competence and need for further training among registered nurses in somatic hospital wards in Sweden: a cross-sectional survey. BMC Nurs.

[25] Willman A, Bjuresäter K, Nilsson J (2020). Newly graduated nurses’ clinical competencies and need for further training in acute care hospitals. J Clin Nurs.

[26] Roth C, Wensing M, Breckner A, Mahler C, Krug K, Berger S (2022). Keeping nurses in nursing: a qualitative study of German nurses’ perceptions of push and pull factors to leave or stay in the profession. BMC Nurs.

[27] Henni SH. Integration of advanced geriatric nurses: a mixed methods study of role and scope of practice. Doctoral thesis. University of Oslo; 2020. https://www.duo.uio.no/bitstream/handle/10852/79857/PhD-Henni-2020.pdf?sequence=1 (accessed 17 November 2023).

[28] Åbo Akademi University. Master’s Degree Programme in Advanced Practice Nursing. https://www.abo.fi/en/study-programme/masters-degree-programme-in-advanced-practice-nursing/ Accessed 15 Nov 2023.

[29] Aarhus University. Sygepleje Kandidatuddannelse [Nursing Master’s Degree Programme]. https://kandidat.au.dk/sygepleje Accessed 25 Oct 2023.

[30] Von Elm E, Altman DG, Egger M, Pocock SJ, Gøtzsche PC, Vandenbroucke JP (2014). The strengthening the reporting of Observational studies in Epidemiology (STROBE) Statement: guidelines for reporting observational studies. Int Surg J.

[31] University of Oslo. *Short introduction to Nettskjema*. https://www.uio.no/english/services/it/adm-services/nettskjema/about-nettskjema.html. Accessed 14 May 2023.

[32] Nilsson J, Engström M, Florin J, Gardulf A, Carlsson M (2018). A short version of the nurse professional competence scale for measuring nurses’ self-reported competence. Nurse Educ Today.

[33] Nilsson J, Carlsson M, Johansson E, Egmar A-C, Florin J, Leksell J (2014). Nursing education in a globalized world: nursing students with international study experience report higher competence at graduation. Open J Nurs.

[34] Leksell J, Gardulf A, Nilsson J, Lepp M (2015). Self-reported conflict management competence among nursing students on the point of graduating and registered nurses with professional experience. J Nurs Educ Pract.

[35] Theander K, Wilde-Larsson B, Carlsson M, Florin J, Gardulf A, Johansson E (2016). Adjusting to future demands in healthcare: curriculum changes and nursing students’ self-reported professional competence. Nurse Educ Today.

[36] Nilsson J, Johansson E, Carlsson M, Florin J, Leksell J, Lepp M (2016). Disaster nursing: self-reported competence of nursing students and registered nurses, with focus on their readiness to manage violence, serious events and disasters. Nurs Educ Pract.

[37] Gardulf A, Florin J, Carlsson M, Leksell J, Lepp M, Lindholm C (2019). The Nurse Professional competence (NPC) scale: a tool that can be used in national and international assessments of nursing education programmes. Nord J Nurs Res.

[38] Nilsson J, Gardulf A, Lepp M. (2016). Process of translation and adaptation of the Nurse Professional Competence (NPC) Scale. J Nurs Educ Pract. 2016:6(1):100-3; 10.5430/jnep.v6n1p100

[39] Skaug EA, Ekman S, Kirchhoff JW. Oversetting, tilpasning og testing av the nurse professional competence scale [Translation, adaptation and testing of the Nurse Professional competence Scale]. Nord Nurs Res. 2020;10(3). 10.5430/jnep.v6n1p100.;164– 75.

[40] Sullivan GM, Feinn R (2012). Using effect size—or why the *P* value is not enough. J Grad Med Educ.

[41] George D, Mallery P (2021). IBM SPSS statistics 27 step by step: a simple guide and reference.

[42] Serafin L, Strząska-Kliś Z, Kolbe G, Brzozowska P, Szwed I, Ostrowska A, et al. The relationship between perceived competence and self-esteem among novice nurses–a cross-sectional study. Ann Med. 2022;54(1). 10.1080/07853890.2022.2032820.;484– 94. 10.1080/07853890.2022.2032820 PMC8843132 35132927

[43] Gardulf A, Nilsson J, Florin J, Leksell J, Lepp M, Lindholm C (2016). The Nurse Professional competence (NPC) scale: self-reported competence among nursing students on the point of graduation. Nurse Educ Today.

[44] Henni S, Kirkevold M, Antypas K, Foss C (2018). The role of advanced geriatric nurses in Norway: a descriptive exploratory study. Int J Older People Nurs.

[45] Wu M-J, Zhao K, Fils-Aime F (2022). Response rates of online surveys in published research: a meta-analysis. Comput Hum Behav.

[46] Van de Mortel TF (2008). Faking it: social desirability response bias in self-report research. Aust J Adv Nurs.

[47] Cook CE, Wright A, Wittstein J, Barbero M, Tousignant-Laflamme Y (2021). Five recommendations to address the limitations of patient-reported outcome measures. J Orthop Sports Phys Ther.

[48] World Medical Association (2013). Declaration of Helsinki: ethical principles for medical research involving human subjects. JAMA.

